# Effects of Different Tissue Flossing Applications on Range of Motion, Maximum Voluntary Contraction, and H-Reflex in Young Martial Arts Fighters

**DOI:** 10.3389/fphys.2021.752641

**Published:** 2021-10-15

**Authors:** Miloš Kalc, Samo Mikl, Franci Žökš, Matjaž Vogrin, Thomas Stöggl

**Affiliations:** ^1^Institute of Sports Medicine, Faculty of Medicine, University of Maribor, Maribor, Slovenia; ^2^Department of Orthopaedics, University Medical Centre Maribor, Maribor, Slovenia; ^3^Department of Sport and Exercise Science, University of Salzburg, Salzburg, Austria; ^4^Athlete Performance Center, Red Bull Sports, Thalgau, Austria

**Keywords:** vascular occlusion, motoneuron excitability, ankle, joint, muscle, elastic band

## Abstract

The purpose of this study was to investigate the effects of tissue flossing applied to the ankle joint or to the calf muscles, on ankle joint flexibility, plantarflexor strength and soleus H reflex. Eleven young (16.6 ± 1.2 years) martial arts fighters were exposed to three different intervention protocols in distinct sessions. The interventions consisted of wrapping the ankle (ANKLE) or calf (CALF) with an elastic band for 3 sets of 2 min (2 min rest) to create vascular occlusion. A third intervention without wrapping the elastic band served as a control condition (CON). Active range of motion for ankle (AROM), plantarflexor maximum voluntary contraction (MVC), and soleus H reflex were assessed before (PRE), after (POST), and 10 min after (POST10) the intervention. The H reflex, level of pain (NRS) and wrapping pressure were also assessed during the intervention. Both CALF and ANKLE protocols induced a significant drop in H reflex during the intervention. However, the CALF protocol resulted in a significantly larger H reflex reduction during and after the flossing intervention (medium to large effect size). H reflexes returned to baseline levels 10 min after the intervention in all conditions. AROM and MVC were unaffected by any intervention. The results of this study suggest that tissue flossing can decrease the muscle soleus H reflex particularly when elastic band is wrapped around the calf muscles. However, the observed changes at the spinal level did not translate into higher ankle joint flexibility or plantarflexor strength.

## Introduction

Martial arts are dynamic activities where the athletes require muscular power, strength, and joint flexibility (Bridge et al., 2014). Static stretching has largely been demonstrated to be an effective method to increase flexibility around a joint (Opplert and Babault, 2018); however, it significantly reduces maximal voluntary strength, which could originate from various neural and peripheral mechanisms (Power et al., 2004; Opplert et al., 2016). The importance of generating high dynamic strength and flexibility is functionally important to support the technical demands in martial arts (Bridge et al., 2014). Thus, static stretching should be used carefully or even avoided during warm-up to prevent subsequent potentially deleterious effects on muscular performance. In the last decade, the interest has focused on the effects of different stretching modalities such as dynamic stretching or foam rolling, which have the potential to increase joint flexibility and maintain muscle power (Opplert and Babault, 2018). Tissue flossing is a novel method, which consists of wrapping part of a limb with a thick elastic band, thus increasing pressure and producing vascular occlusion in a part of that limb distal from the band. Some studies have recently recognized the value of this technique as a way of increasing joint range of motion (ROM) while maintaining or even increasing muscle strength and power (Driller and Overmayer, 2017).

In clinical practice as well as in the current published literature, there are two main band application modes: the joint technique, where the elastic band is wrapped around a joint, and the muscle technique, where the band is wrapped around the muscles of the limb (Konrad et al., 2021). To the best of our knowledge, the effects of tissue flossing on joint ankle ROM and torque of the plantarflexors using the joint and muscle technique have never been compared in a single study on the same pool of participants.

Studies investigating the effects of both techniques have found conflicting results. Studies where the elastic band was wrapped around the ankle joint of recreationally trained individuals, reported an increase in ROM (Driller and Overmayer, 2017), a decrease in muscle tone (Vogrin et al., 2020b), as well as positive effects on muscle strength, jump and sprint performance (Driller et al., 2017; Driller and Overmayer, 2017). However, tissue flossing seems to be less effective when applied to athletes (Mills et al., 2020). The effects of tissue flossing when wrapped around the muscle have produced ambiguous results. Konrad et al. (2021) as well as Vogrin et al. (2020a) wrapped the thigh, reporting an increase in maximal isometric torque of the knee extensors, but no increase in ROM measured by the active straight leg raise (ASLR) test. In contrast, Kaneda et al. (2020) reported a substantial increase in ASLR. Gorny and Stöggl (2018) lacked to find any effect of tissue flossing on DOMS (Delayed Onset Muscle Soreness) reduction.

Despite the increased interest in this field, only a handful of studies investigated the physiological mechanisms responsible for the observed functional changes in ROM and MVC (Konrad et al., 2021). There are different possible mechanisms of action involved in the observed increase in ROM: Kaneda et al. (2020) reported an increased passive torque at the end of the ROM after tissue flossing, which might indicate higher tolerance to stretch. Moreover, the increased ROM was not accompanied by changes in muscle stiffness (Kaneda et al., 2020; Vogrin et al., 2020a). The effects of tissue flossing on ROM can be compared to those elicited via self-myofascial release, which is created by applying pressure on muscles and fascia using foam rollers. Even though the physiological mechanisms responsible for the effects of foam rolling are still part of a scientific debate, the mechanisms can be divided into mechanical, focused on the alteration of the fascia (Schleip and Müller, 2013) and neurophysiological (Schleip, 2003). In this regard, it has been suggested that pressure applied by the tissue flossing on muscle, skin, and fascia could impact fluid viscosity, which could lead to less resistance to movement (Konrad et al., 2021).

Increased muscular strength and power after tissue flossing could be explained by increased sympathetic outflow and a facilitation of the short-latency stretch reflex (Konrad et al., 2020). It is well known that afferent signals from muscle spindles contribute to different voluntary muscle contractions (Macefield et al., 1991), and thus an increase in spinal excitability can induce an increase in performance. Furthermore, Vogrin et al. (2020a) reported a reduction in contraction time in the rectus femoris after tissue flossing, which was interpreted as neuromuscular potentiation.

Besides the effects caused by pressure on muscle, skin and fascia, tissue flossing induces vascular occlusion, which can be compared with ischemic preconditioning (Driller and Overmayer, 2017). Ischemic preconditioning is a technique where sub-lethal local acute ischemia is induced by arterial occlusion. It is thought that ischemic preconditioning can prevent or attenuate future ischemic reperfusion injuries in the exposed tissue (Murry et al., 1986). Recently, this method was tested in other applications, resulting in enhanced exercise performance (Cocking et al., 2018), delayed time to fatigue (Halley et al., 2018) and improvements in postural control (Cherry-Allen et al., 2015). Due to differences in protocol conditions and durations between ischemic preconditioning studies, is it still unclear if ischemic preconditioning induces an increase or depression of neuromuscular performance. Both methods, ischemic preconditioning and tissue flossing, create vascular occlusion, thus limiting oxygen availability to the wrapped body part. This is borne out by a recent study by Pavlů et al. (2021) that confirmed a drastic drop in blood flow during 2 min flossing application.

Oxygen availability as well as afferent inputs from muscle spindles and other pressure-sensitive receptors, play an essential role in the function of the central nervous system (CNS). A reduction in oxygen supply (hypoxia) quickly affects neural mechanisms, especially their metabolic requirements and excitability (Neubauer and Sunderram, 2004). On the other hand, afferent signaling from mechanoreceptors might stimulate the nervous system and thereby lead to reduced muscle stiffness (Schleip, 2003). There is some evidence that pressure exerted by foam rolling treatment decreases neural modulation of spinal excitability (Young et al., 2018). Studies suggest that changes in the CNS are peripherally mediated central effects via III and IV afferents from within the muscle (Verges et al., 2012), skin and fascia (Schleip, 2003). At the spinal level, neural impairment can be partially linked to the inhibition or excitability of the alpha motoneuron pool (Garland and McComas, 1990). We, therefore, believe it is worth exploring the effects of tissue flossing on the excitability of the motoneuron pool employing a widely accepted technique like the H reflex (Zehr, 2002). The H reflex is electrically evoked and considered the analog of the stretch reflex since both share the same monosynaptic reflex pathway (Zehr, 2002). However, the H reflex bypasses the muscle spindle; thus, its amplitude depends not only on motoneuron excitability (Schieppati, 1987) but also on the level of presynaptic inhibition affecting the Ia afferents (Knikou, 2008). Earlier literature describes the effects of systemic hypoxia on H reflex in ambiguous terms, showing increased (Delliaux and Jammes, 2006), decreased (Willer et al., 1987), or unchanged H reflex amplitude (Kayser et al., 1993). Tissue flossing using joint and muscle wrapping techniques might have different effects on neuromuscular parameters in respect of unique receptors present in both the muscle and joint capsule tissue (Taylor, 2009).

Based on previous studies, we hypothesize an increase in ankle joint ROM after the joint application, an increase in plantarflexion MVC after the muscle application and a higher reduction of the H-reflex using the muscle application compared to the joint application. Therefore, the aim of the study was to investigate the effects of tissue flossing applied to the ankle joint or to the calf muscles on ankle ROM, plantarflexor MVC and muscle soleus H reflex.

## Materials and Methods

### Study Design

**FIGURE 1:**
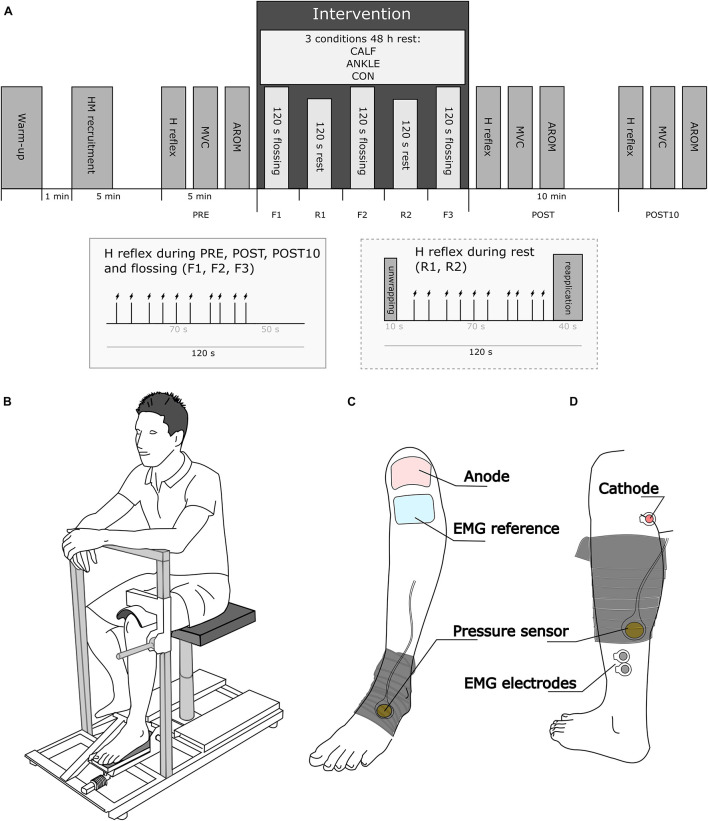
**(A)** Graphical representation of the study design. AROM and MVC were assessed before (PRE) and after the intervention (POST and POST10). The H reflex was assessed during the flossing and control intervention (F1, F2, F3) and in the rest periods between flossing and control interventions (R1, R2). The small box captions represent the timeline of nerve stimulation during PRE, F1, F2, F3, POST, POST10, and R1, R2, respectively. **(B)** Schematic of the position of the subject during the neuromuscular assessment. **(C)** Flossing band wrapping during the ANKLE condition; **(D)** flossing band during the CALF condition. **(C,D)** Show the placement of the pressure sensor probe, EMG and stimulation electrodes.

The present study followed a cross-over repeated measures design, where each participant was randomly exposed to three intervention protocols, one per visit. At each visit, following warm-up and pretest assessments, an elastic band (Medical Flossing Band, Germany, 1.3 mm thick, 50 mm wide, 150 mm long) was used to wrap the ankle (ANKLE condition) or calf (CALF condition) of the left leg. The third visit without elastic band wrapping was used as the control condition (CON). During each visit, participants underwent the same assessments prior to (PRE), immediately after (POST) and 10 min after (POST10) the intervention (floss band application or control). The following lower-leg assessments were conducted: (i) muscle soleus H-reflex (HM); (ii) plantarflexion MVC; (iii) active ankle range of motion (AROM). In addition, H-reflexes were elicited while the band was applied (F1, F2, F3) and during the rest period between applications (R1, R2) (see Figure 1A). The same procedure was applied in the CON condition without band application. There were 48-h between consecutive visits to avoid between-session influences.

### Participants

Eleven young elite non-Olympic martial arts fighters (Biological sex: 5 men, 6 women, Age: 16.6 ± 1.2 years, Weight: 62.3 ± 7.9 kg, Height: 171.3 ± 9.0 cm) participated in this study (power 0.6). Participants undertook regular training (Weekly training session: 7.7 ± 5.0; Weekly training volume: 9.3 ± 1.0 h) and participated in international and domestic Taekwondo and Kickbox tournaments. Recent competitive achievements were 3 World and 4 European champions in their age group; 6 fighters were classified in the world top 5 rank in their age group. All participants were free of acute locomotor, nerve, or known cardiovascular and metabolic diseases. Prior to the first visit, the participants completed a modified risk assessment questionnaire (Kacin et al., 2015), to exclude possible safety risks while inducing vascular occlusion. Among the exclusion criteria were family history of clotting disorders (e.g., lupus, hemophilia, high platelets), level 1 hypertension (SAP ≥ 140 mmHg), hypertension (SAP 120–140 mmHg), history of deep vein thrombosis, pulmonary embolism, history of hemorrhagic or thrombotic stroke, smokers, medication including the contraceptive pill, history of nerve injury (including back or neck injury), history of injury to arteries or veins, diabetes, metalwork *in situ*, undiagnosed groin or calf pain, compartment syndrome, surgery within the previous 4 weeks, a journey lasting more than 4 h or a flight in the previous 7 days and any other medical conditions including a history of synovitis.

The participants were familiarized with the experimental procedure and their voluntary cooperation was confirmed by written informed consent on the first testing day. The study was approved by the Research Ethics Committee of the University Medical Centre Maribor, Slovenia. All procedures were performed according to the Declaration of Helsinki.

### Assessment Procedures

#### Maximum Voluntary Contraction Assessment

To assess the MVC of ankle plantarflexors, participants were instructed to sit on an ankle dynamometer equipped with a force sensor (S2P, Ljubljana, Slovenia) with a sampling rate of 1,000 Hz. Their hips, knees and ankles were flexed at 90°. The lateral malleolus of the tibia was aligned with the dynamometer’s axis of rotation and plantarflexion movement was restricted using the dynamometer fixation system, which uses a rigid brace pressed to the thigh, just above the knee joint (Figure 1B). At the beginning of each visit, the participants were familiarized with the MVC technique and asked to contract their plantarflexors seven times for 5 s (30 s between contractions). They were instructed to exert a medium effort (subjectively defined) at the first contraction and progressively increase effort at every consecutive contraction until reaching a maximal contraction at the last trial. This procedure also served as a warm-up. At each time point (PRE, POST, POST10), the participants were instructed to progressively contract their plantarflexors until the maximum torque was reached (within 2 s approximately), maintaining the maximum isometric contraction for approximately 4 s. Participants were verbally encouraged to perform the tests with maximal effort. No visual feedback was provided (Verges et al., 2009). Two repetitions with 30 s rest were permitted at each time point.

#### Range of Motion Assessment

The ROM of the left ankle was assessed using a G-Walk (BTS, Bioengineering, Italy) digital goniometer with a sampling rate of 1,000 Hz. Participants were instructed to lie supine on a (standard) therapeutic table, with arms alongside the body. In order to permit unrestricted ankle motion, the left ankle extended past the edge of the table, with the edge supporting the lower leg just above the malleoli on the distal third. In addition, a cushion was placed below the knee joint allowing 30° knee flexion. The contralateral leg was flexed in the hip and knee and in contact with the table. The goniometer was placed just below the lateral malleolus of the ankle, with the *y*-axis lined up through the lateral aspect of the fibula and the *x*-axis lined up with the 5th metatarsophalangeal joint. The goniometer was attached to the skin using bandaging tapes and additionally fixed in position with an elastic bandage. The position of the goniometer at the PRE time point was marked using a skin marker to allow accurate repositioning at POST and POST10 time points. At each measuring time (PRE, POST, and POST10), the participants were instructed to slowly (within 2 s approximately) extend the left ankle (plantarflexion) and maintain that position for approximately 1 s before slowly flexing the ankle (dorsal flexion). Once maximum dorsiflexion was reached, participants maintained the final position for approximately 1 s before slowly (approx. 2 s) returning to the starting position. The procedure was repeated twice for each time point.

#### Surface Electromyography

Participants were prepared for surface electromyographic (EMG) recording of the soleus muscle (SOL) using a standard procedure: the skin was shaved and slightly rubbed using an abrasive paste. Electrodes (Covidien 24 mm, Walpole, Massachusetts, United States) for recording the H reflex from the SOL muscle were placed in a standard bipolar configuration at an interelectrode distance of 20 mm. The reference electrode (50 × 100 mm, 00734, Compex, Guildford, Surrey, United Kingdom) was placed over the tuberositas tibiae. The EMG signal was collected using a PowerLab data acquisition toolbox and LabChart 8 software (both ADInstruments, Bella Vista, New South Wales, Australia) at 4,000 Hz sampling frequency and filtered using a 10–500 Hz band-pass filter.

#### Electrical Stimulation

All stimulations were performed with the participants in the same position for measuring MVC. H reflex and respective M waves measured in SOL were elicited by a custom-built, constant current high voltage electrical stimulator (NeoStim 1, FE Furlan, Ljubljana, Slovenia) delivering single rectangular electrical impulses (1 ms) to the tibial nerve in 5–8 s pseudo-random interstimulus intervals. This interstimuli interval minimized the possibility of post-activation depression (Zehr, 2002; Burke, 2016). The anode (50 × 90 mm, MyoTrode PLUS, Globus, Italy) was placed over the patella and the cathode (Covidien 24 mm, Walpole, Massachusetts, United States) was placed in the popliteal fossa at the position that provided the greatest M wave at 20 mA intensity. Such high intensity was used only to detect the best stimulation position. The cathode position was marked with a skin marker and additionally fixed using an elastic band. In the preparatory phase of each visit (before PRE time point), an H reflex—M wave recruitment (HM recruitment) curve was assessed starting at 10 mA, increasing the stimulation intensity by 1 mA every successive stimulation until the H reflex clearly reached its descending phase. Stimulation intensities were then increased in 5 mA steps until a maximal M wave was reached (Mmax). Stimulation intensity during experimental measuring was adjusted to obtain a value where the H reflex would fall on the ascending part of the HM recruitment curve with an M wave value of approximately 10% Mmax amplitude (M10). 10 stimuli at M10 intensity were delivered at each time point (PRE, F1, R1, F2, R2, F3, POST, POST10). Particular attention was made to starting the stimulation protocol within 5 s from the start of each time point. The intensity was slightly adjusted during the experiment to elicit a consistent M10 amplitude.

#### Level of Pain

The level of pain was assessed using an 11-point numerical rating scale (NRS), where 0 indicates “no pain” and 10 indicates the “worst imaginable pain.” 60 s after the floss band was applied, participants were instructed to choose a single number from the scale that best indicated their level of pain (Hjermstad et al., 2011). The level of pain was not assessed during the CON condition.

#### Kikuhime Pressure Control Sensor Measurement

A flat balloon-like pressure control sensor (35 mm in diameter; Kikuhime, TT Meditrade, Sorø, Denmark) was used to control the pressure exerted by the floss band to the wrapped ankle or calf. This device represents a validated (ICC = 0.99, CV = 1.1%) and reliable (CV = 4.9%) tool to be used in the sports medicine setting (Brophy-Williams et al., 2013). The pressure control sensor was fixed with a tape patch over the tube of the sensor. During the CALF visit, the sensor was placed on the triceps surae aponeurosis just below the gastrocnemius bulk, while during the ANKLE visit, the sensor was placed on the cuneiform bones. To ensure precise pressure measurements, the tube attaching the sensor to the digital display was always set facing proximally toward the knee while the sensor itself was set toward the floss band and progressively compressed by the band application (Figures 1C,D).

### Floss Band Application

Following pre-tests, participants were instructed to sit on the ankle dynamometer. To facilitate wrapping and unwrapping of the floss band, they were asked to extend their left knee, placing the lower part of the leg on a pillow. The leg position (wrapping position) was slightly different between CALF and ANKLE conditions. During the ANKLE visit, the pillow was placed at the middle-third of the lower leg on the medial gastrocnemius muscle, while during the CALF and CON visits, the pillow supporting the leg was placed in the distal third of the lower leg, just above the calcaneus. In visits where banding was applied (ANKLE and CALF), the floss band, comprising a thick latex elastic band, was wrapped around the ankle joint (ANKLE) or the calf muscles (CALF). The floss band was applied by two physical therapists (SM and FZ) with 5 years of experience of tissue flossing methods. In both flossing visits, a simple bandaging technique was used: the elastic band was first pulled and then wrapped in a circular motion around the limb moving proximally toward the knee in a progressive way. Each subsequent wrap overlapped the previous by approximately 50%, before fixing the last part of the band (approx. 5 cm) beneath the final wrap. In the CALF visit, the first wrap started at the middle of an imaginary line between the external malleolus and the head of the fibula, approximately 5 cm above the position of the EMG electrodes. In the ANKLE visit, the first wrap started at the middle of the metatarsal bone (distal of the talus). The calcaneus and the Achilles tendon were also wrapped with the elastic band. In both visits, the floss band covered approximately 15 cm of the limb when fully applied. The physical therapists were instructed to apply the flossing band by pulling the band at 100% extension as they would do in regular clinical practice. Wrapping pressure was monitored during each application and the floss band was reapplied if pressure was below 150 mmHg, which happened in one case. The wrapping procedure took approximately 40 s. As soon as the last part of the band was fixed, the subject was asked to return to the H-reflex assessment position. After 120 s participants were instructed to extend their legs and the floss band was unwrapped. As soon the band was unwrapped (approximately 10 s), participants were instructed to return to the H-reflex assessment position for approximately 70 s and then to the wrapping position for the band to be reapplied. This procedure was repeated three times (3 sets of 120 s flossing followed by 80 s rest and 40 s of reapplication).

### Data Processing

MVC and ROM data were processed using the RcppRoll package (Ushey, 2018) within the R language environment (R Core Team, 2020). MVC data was first processed using a rolling average filter (0.2 s). The highest peak torque across the two trials for the same assessment time point (PRE, POST, or POST10) was taken for further analysis. ROM data was first processed using a rolling average filter (0.1 s). The highest plantarflexion and dorsiflexion angles across two trials for the same assessment time point was taken for further analysis. The AROM parameter was computed by adding the plantarflexion and dorsiflexion angle data.

**FIGURE 2:**
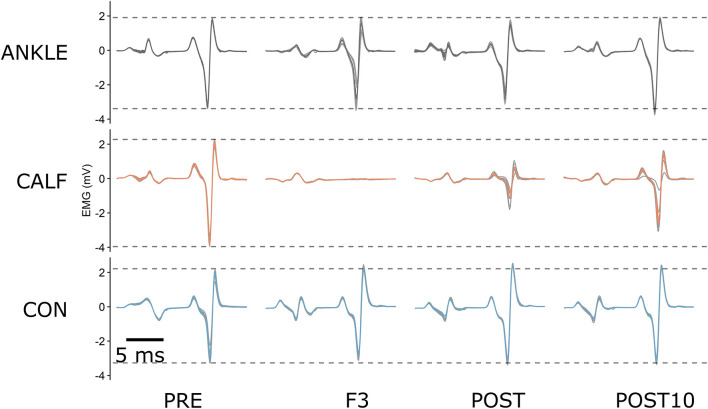
An example of raw H reflex data for one representative participant at PRE, during F3, POST and POST10 time points. The colored lines represent the average of 9 consecutive signals at each time point. ANKLE, CALF, and CON conditions are represented by gray, orange and blue lines, respectively. Dashed lines represent baseline levels for each condition.

10 H reflexes were elicited at each assessment time point and checked for consistency. The first stimuli at each timepoint were used to adjust the stimulation intensity and were discarded during the data processing (discarded: 264). Moreover, the remaining M waves with peak to peak amplitudes differing more than ± 2 SDs (Budini et al., 2017) from the baseline of each visit were discarded (elicited: 2,376; discarded: 40 = 1.7%). The data was normalized to the maximal *M*-values. The average value of all H reflexes within a time point was used for further analysis as the HM parameter (Figure 2).

### Statistical Analysis

Data was analyzed using the R (version 3.5.4) programming language (R Core Team, 2020). Normal distribution was verified using the Shapiro–Wilk test for small samples across all parameters. A 3 × 3 two-way repeated-measures ANOVA using the Afex package (Singmann et al., 2020) was performed to determine the effect of different treatments (CON, CALF, ANKLE) and time (PRE, POST, POST10) on MVC and AROM. A 3 × 8 two-way repeated-measures ANOVA was performed to determine the effect of different treatments (CON, CALF, ANKLE) and time (PRE, POST, POST10, F1, F2, F3R1, R2) on the H-reflex, M wave (M10) and stimulation intensity (STIMint). In addition, a 2 × 3 two-way repeated-measures ANOVA was performed to determine the effects of 2 flossing conditions (CALF, ANKLE) over the three time points where the elastic band was applied (F1, F2, F3) on NRS and pressure parameter. The assumption of sphericity was assessed using the Mauchly test. Whenever the assumption of sphericity was violated, the degrees of freedom were corrected using the Greenhouse-Geisser correction (GGe). *Post hoc* tests were performed as pairwise comparisons using Tukey’s adjustment within the Emmeans package (Lenth et al., 2020) to determine the differences between single treatments at different time points. Standardized changes in the mean of each measure were used to assess magnitudes of effects and were calculated using Cohen’s *d* and then interpreted using thresholds of 0.2, 0.5, 0.8 for small, moderate and large effects, respectively (Batterham and Hopkins, 2006). An effect size of ± 0.2 was considered the smallest worthwhile effect, with an effect size of < 0.2 considered to be trivial. The effect was considered unclear if its 95% confidence interval overlapped the thresholds for small positive and small negative effects (Batterham and Hopkins, 2006). Statistical significance was set at *p* < 0.05 for all analyses. Correlations between H-reflex relative changes from baseline and pressure were computed using the Pearson correlation coefficient.

## Results

Descriptive statistics (mean and SD) for ASLR, MVC, and H-reflex data are given in Table 1 and for NRS and pressure in Table 2. All observed variables were normally distributed. Cohen’s *d* effect sizes for comparisons of all measures (POST, POST10) to pre-test values are given in Table 3.

**TABLE 1:** Descriptive statistics (mean ± SD).

Variable	ANKLE	CALF	CON
***AROM (*°*)***			
PRE	66.4 ± 9.2	63.3 ± 11.6	66.3 ± 8.8
POST	67.5 ± 11.4	63.7 ± 12.2	65.5 ± 10.0
POST10	65.9 ± 10.7	63.2 ± 13.0	63.8 ± 10.5
***MVC (Nm)***			
PRE	115.0 ± 15.1	113.6 ± 14.3	111.2 ± 16.2
POST	117.6 ± 19.4	109.7 ± 15.5	108.7 ± 16.3
POST10	116.4 ± 17.0	113.6 ± 19.1	117.9 ± 19.8
***H (%Mmax)***			
PRE	49.4 ± 26.9	54.8 ± 22.0	53.2 ± 19.9
F1	30.5 ± 29.6	21.7 ± 20.6	45.0 ± 23.4
R1	44.5 ± 31.7	43.6 ± 23.1	47.8 ± 21.3
F2	31.7 ± 29.9	20.1 ± 22.3	45.2 ± 22.0
R2	45.7 ± 32.0	38.8 ± 23.1	45.1 ± 20.5
F3	34.6 ± 29.6	22.8 ± 19.6	47.7 ± 22.2
POST	46.0 ± 31.8	37.5 ± 23.6	46.2 ± 21.6
POST10	46.7 ± 33.8	48.5 ± 26.1	45.3 ± 25.9
***M10 (%Mmax)***			
PRE	8.0 ± 4.8	10.8 ± 6.1	11.8 ± 5.8
F1	7.5 ± 4.3	10.9 ± 6.4	10.3 ± 4.9
R1	7.6 ± 3.9	10.5 ± 5.1	10.5 ± 5.0
F2	8.0 ± 4.8	11.6 ± 7.0	10.6 ± 5.3
R2	7.6 ± 4.2	10.4 ± 5.8	11.3 ± 6.3
F3	7.7 ± 4.1	11.0 ± 6.5	10.6 ± 5.0
POST	8.3 ± 4.8	11.1 ± 6.3	10.4 ± 4.9
POST10	8.0 ± 4.4	11.0 ± 6.6	10.5 ± 5.2
***STIMint (mA)***			
PRE	25.0 ± 7.3	26.1 ± 9.1	30.5 ± 16.4
F1	25.6 ± 8.9	27.1 ± 8.7	31.0 ± 14.2
R1	24.4 ± 8.6	27.4 ± 9.6	30.4 ± 13.8
F2	23.4 ± 9.1	25.1 ± 7.3	29.9 ± 13.9
R2	23.7 ± 9.5	26.0 ± 9.0	29.9 ± 13.7
F3	24.0 ± 9.0	25.0 ± 7.3	29.8 ± 13.0
POST	23.0 ± 8.5	25.3 ± 8.4	29.5 ± 13.2
POST10	21.2 ± 8.1	24.2 ± 9.3	27.5 ± 13.3

*ANKLE, elastic band wrapped around the ankle joint; CALF, elastic band wrapped around the calf muscles; CON, control intervention; PRE, assessment before intervention; POST, assessment after the intervention; POST10, assessment 10 min after the intervention; F1, F2, F3, assessments during first, second and third band application; R1, R2, assessment during first and second rest between band applications; ROM, ankle range of motion from maximal plantarflexion to maximal dorsiflexion; MVC, plantarflexion maximum voluntary contractions; H, soleus H reflex expressed as a percentage of maximal M wave; M10, soleus M wave expressed as a percentage of maximal M wave; STIMint, stimulation intensity.*

**TABLE 2:** Descriptive statistics (mean ± SD).

Variable	ANKLE	CALF
***NRS***		
F1	4.4 ± 1.4	4.6 ± 1.5
F2	4.1 ± 1.5	4.3 ± 1.5
F3	5.0 ± 1.4	4.1 ± 1.1
***Pressure (mmHg)***		
F1	290.3 ± 48.5	226.2 ± 57.6
F2	294.8 ± 51.3	238.4 ± 48.4
F3	296.7 ± 64.3	236.2 ± 51.9

*ANKLE, elastic band wrapped around the ankle joint; CALF, elastic band wrapped around the calf muscles; F1, F2, F3, assessments during first, second and third band application; NRS, numerical rating scale for pain assessment during flossing; Pressure, pressure exerted by the elastic band.*

**TABLE 3:** Cohen’ d effect size and 95% confidence intervals.

Variable	ΔANKLE – ΔCON	ΔCALF – ΔCON	ΔCALF – ΔANKLE
	effect size ± 95%CI	effect size ± 95%CI	effect size ± 95%CI
***ROM (*°*)***			
POST	0.5 ± 0.9 *unclear*	0.3 ± 0.8 *unclear*	0.2 ± 0.9 *unclear*
POST10	0.4 ± 0.8 *unclear*	0.5 ± 0.8 *unclear*	0.1 ± 0.7 *unclear*
***MVC (Nm)***			
POST	0.5 ± 0.7 *unclear*	−0.1 ± 1.0 *unclear*	−0.6 ± 0.8 *medium*
POST10	−0.4 ± 0.4 *small*	−0.4 ± 0.8 *unclear*	−0.1 ± 0.7 *unclear*
***H (% Mmax)***			
F1	−0.6 ± 0.7 *medium*	−1.4 ± 1.3 *large*	−0.7 ± 0.7 *medium*
R1	0.0 ± 0.4 *unclear*	−0.4 ± 0.9 *unclear*	−0.4 ± 0.8 *unclear*
F2	−0.6 ± 0.8 *unclear*	−1.5 ± 1.4 *large*	−0.8 ± 0.7 *large*
R2	0.3 ± 0.6 *unclear*	−0.5 ± 0.8 *unclear*	−0.7 ± 0.7 *medium*
F3	−0.6 ± 0.8 *medium*	−1.9 ± 1.3 *large*	−0.9 ± 0.5 *large*
POST	0.3 ± 0.5 *small*	−0.6 ± 1.0 *unclear*	−0.7 ± 0.9 *medium*
POST10	0.4 ± 0.5 *small*	0.1 ± 0.9 *unclear*	−0.2 ± 0.6 *unclear*

*ANKLE, elastic band wrapped around the ankle joint; CALF, elastic band wrapped around the calf muscles; CON, control intervention; POST, assessment after the intervention; POST10, assessment 10 min after the intervention; F1, F2, F3, assessments during first, second and third band application; R1, R2, assessment during first and second rest between band applications; ROM, ankle range of motion from maximal plantarflexion to maximal dorsiflexion; MVC, plantarflexion maximum voluntary contractions; H, soleus H reflex expressed as a percentage of maximal M wave; 95%CI, 95% confidence interval.*

The analysis of NRS revealed that there was not a statistically significant interaction between the effect of intervention type and time [*F*_(2, 20)_ = 2.17, *P* = 0.141, η*_*p*_*^2^ = 0.178]. Simple main effect analysis showed that time [*F*_(2, 20)_ = 0.31, *P* = 0.735, η*_*p*_*^2^ = 0.030] and intervention type [*F*_(2, 20)_ = 2.17, *P* = 0.141, η*_*p*_*^2^ = 0.178] did not have a statistically significant effect on NRS.

The analysis of pressure revealed that there was not a statistically significant interaction between the effect of intervention type and time [*F*_(2, 20)_ = 0.26, *P* = 0.771, η*_*p*_*^2^ = 0.026]. Simple main effect analysis showed that intervention type did have a statistically significant effect on pressure [*F*_(1, 10)_ = 21.05, *P* < 0.001, η*_*p*_*^2^ = 0.678]. Simple main effect analysis showed that time did not have a statistically significant effect on pressure [*F*_(2, 20)_ = 1.47, *P* = 0.254, η*_*p*_*^2^ = 0.128].

The analysis of AROM revealed that there was not a statistically significant interaction between the effect of intervention type and time [*F*_(4, 40)_ = 0.64, *P* = 0.635, η*_*p*_*^2^ = 0.060]. Simple main effect analysis showed that intervention type did have a statistically significant effect on AROM [*F*_(2, 20)_ = 4.19, *P* = 0.030, η*_*p*_*^2^ = 0.295]. Simple main effect analysis showed that time did not have a statistically significant effect on AROM [*F*_(2, 20)_ = 1.23, *P* = 0.312, η*_*p*_*^2^ = 0.110]. *Post hoc* tests revealed no statistically significant differences.

The analysis of MVC revealed that there was not a statistically significant interaction between the effect of intervention type and time [*F*_(4, 40)_ = 1.56, *P* = 0.203, η*_*p*_*^2^ = 0.135]. Simple main effect analysis showed that time [*F*_(2, 20)_ = 1.06, *P* = 0.366, η*_*p*_*^2^ = 0.096] and intervention type [*F*_(2, 20)_ = 1.73, *P* = 0.202, η*_*p*_*^2^ = 0.148] did not have a statistically significant effect on MVC.

The analysis of M10 revealed that there was not a statistically significant interaction between the effect of intervention type and time [*F*_(14, 140)_ = 0.89, *P* = 0.568, η_*p*_^2^ = 0.082]. Simple main effect analysis showed that time [*F*_(7, 70)_ = 0.76, *P* = 0.626, η*_*p*_*^2^ = 0.070] and intervention type [*F*_(2, 20)_ = 1.80, *P* = 0.191, η*_*p*_*^2^ = 0.153] did not have a statistically significant effect on M10.

The analysis of STIMint revealed that there was not a statistically significant interaction between the effect of intervention type and time [*F*_(14, 140)_ = 0.29, *P* = 0.995, η*_*p*_*^2^ = 0.028]. Simple main effect analysis showed that time did have a statistically significant effect on STIMint [*F*_(7, 70)_ = 2.74, *P* = 0.014, η*_*p*_*^2^ = 0.215]. Intervention type did not have a statistically significant effect on STIMint [*F*_(2, 20)_ = 1.52, *P* = 0.242, η*_*p*_*^2^ = 0.132].

**FIGURE 3:**
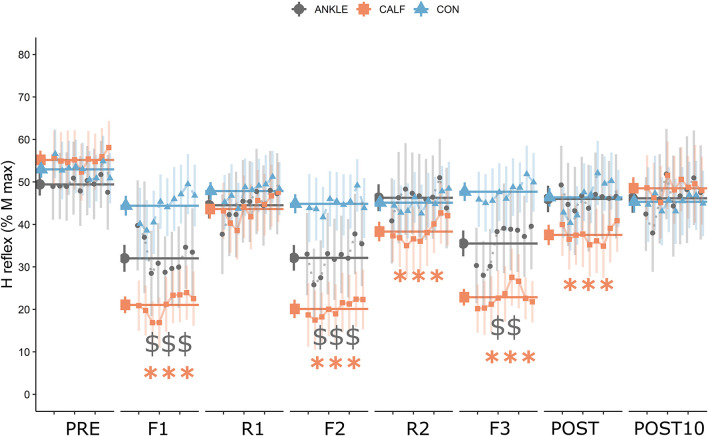
H reflex data expressed as a percentage of Mmax. Orange squares represent data for the CALF condition, gray dots represent data for the ANKLE condition and blue triangles represent data for the CON condition. Larger points and horizontal lines represent the mean and standard error of the average of all H reflex (9 signals) amplitudes in each time point. Small points represent the mean and standard error of each measured H reflex. Statistically significant differences from baseline are represented by asterisks for the CALF condition: ^∗∗∗^*P* < 0.001; and dollars for the ANKLE condition: ^$$^*P* < 0.01, ^$$$^*P* < 0.001.

The analysis of H-reflex revealed that there was a statistically significant interaction between the effect of intervention type and time [*F*_(14, 140)_ = 6.08, *P* < 0.001, η*_*p*_*^2^ = 0.378]. Simple main effect analysis showed that time did have a statistically significant effect on H-reflex [*F*_(7, 70)_ = 11.68, *P* < 0.001, η*_*p*_*^2^ = 0.539]. Simple main effect analysis showed that intervention type did not have a statistically significant effect on H-reflex [*F*_(2, 20)_ = 2.45, *P* = 0.112, η*_*p*_*^2^ = 0.197]. *Post hoc* comparisons revealed significant statistical differences for CALF condition between PRE and F1, F2, R2, F3, and POST measurements. Significant differences were found for the ANKLE condition between PRE, F1, F2, F3. The average value of all H reflexes within a time point was used for further analysis as the HM parameter (Figure 3).

**FIGURE 4:**
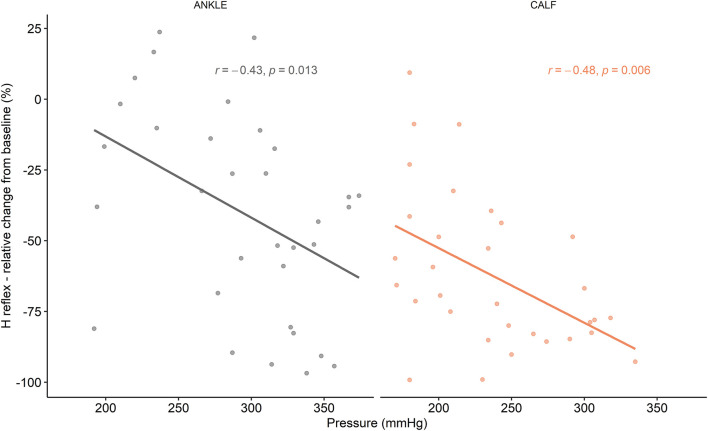
Correlations between H reflex relative changes from baseline and flossing pressure.

A medium, but statistically significant correlation was found between pressure and H-reflex mean relative change from baseline during F1, F2, and F3 timepoints for the CALF [*r*(33) = −0.48, *P* = 0.006] and ANKLE condition [*r*(33) = −0.43, *P* = 0.013] (Figure 4).

## Discussion

The aim of this study was to investigate the effects of tissue flossing applied on the ankle joint or on the calf muscles on the ankle ROM, plantarflexor MVC and SOL H reflex. To the best of our knowledge, this is the first study that compared the effects of the two most common tissue flossing techniques on various neuromuscular parameters.

The main findings of this study were that the H reflex was statistically significantly reduced during flossing in CALF and ANKLE conditions and immediately after flossing in the CALF condition. However, the H reflex was much more affected during the CALF condition compared to the ANKLE condition (*medium* to *large* effect size). The data shows a possible cumulative effect of tissue flossing application on spinal excitability in the CALF condition. In fact, there were statistically significant changes in H reflex at R2 (second rest between band applications) and at POST timepoint. In comparison, H reflex in ANKLE condition returned to baseline levels as soon as the floss band was unwrapped. To the best of our knowledge, this was the first study to investigate the effects of tissue flossing on H reflex; therefore, there were no studies to compare our results to. We have found only a few studies investigating the effect of local acute ischemia on H reflex: Zakutansky et al. (2005) registered a significant decline in Hmax/Mmax ratio (−12%) during and after 5 min of acute ischemia. In comparison, our study revealed a much larger decrease in H reflex during flossing (CALF: −63%; ANKLE: −38%) and after the intervention (−32%) for the CALF condition. In contrast, Mendonca et al. (2020) found no differences in H reflex amplitude and nerve conduction velocity following 60 and 80% arterial occlusion. Significant differences between our work and the aforementioned studies on inducing local acute ischemia could be related to the fact that in our study, the elastic band had been wrapped closer to the EMG electrodes; with acute ischemia being induced by femoral artery occlusion in the proximal part of the thigh. There is some evidence in the literature that changes in neuron excitability threshold is more pronounced closer to the site of ischemic compression (Bostock et al., 1991). This could also partially explain the observed difference between conditions since the CALF application was closer to the EMG electrodes compared to the ANKLE application. In addition to the effects of vascular occlusion induced by ischemia, we should take in consideration the effects of compression on muscles, skin and fascia. It was recently suggested by Young et al. (2018) that high-intensity foam rolling can affect spinal excitability, resulting in a decrease of soleus H reflex up to 58%. A similar reduction in spinal reflexes can be seen during manual massage therapy (Morelli et al., 1990; Goldberg et al., 1992; Behm et al., 2013). We can speculate that the large decrease in H reflex during tissue flossing registered in this study originate from mechanisms related to local ischemia and compression of mechanoreceptors present in the muscles, skin and fascia. The fast modulation of the H reflex observed during flossing and rest periods suggest that highly sensitive and rapidly adapting receptors are responsible for mediating this response.

One of the possible mechanisms explaining the reduction of the H reflex registered in this study is presynaptic inhibition. It has been well documented that this mechanism regulates the amount of neurotransmitters to be released in the Ia-motoneuron synapse regardless of the firing rate of the Ia afferents (Chalmers and Knutzen, 2002). Therefore, it is plausible that ischemia and compression of mechanoreceptors may affect the presynaptic interneurons and downregulate the effect of Ia fibers into motoneurons (Burke et al., 1984). However, even though there are several methods to measure presynaptic inhibition (Knikou, 2008), this study was not designed to do so. Therefore, the effect of tissue flossing on presynaptic inhibition needs to be examined in a future study.

A second explanation for the decrease in H reflex is the possible effect that produces a hyperpolarized motoneuron. There is emerging evidence in the literature that group III and IV afferences play an important role in spinal modulation (Burke et al., 1984). A hyperpolarized motoneuron would result in a less excitable motoneuron pool, requiring more stimulation intensity to induce the same H reflex. However, the mechanism is less probable in this study since there were no differences in stimulation intensity between time points. Group III and IV muscle afferents are also thought to be activated by nociceptive stimuli and are associated with pain perception (McCord and Kaufman, 2010), which could explain differences in the spinal excitability observed in this study. However, there were no differences in the perception of pain between CALF and ANKLE conditions.

Nonetheless, we cannot dismiss the possibility that compression induced by the elastic band could induce reversible mechanical changes in the wrapped tissue. It is unlikely that short-lasting tissue flossing could affect the viscoelastic or mechanical properties of the fascia, since it was demonstrated that forces outside the normal physiological range are required to produce significant release of the fascia (Chaudhry et al., 2008). Compression of muscle tissue, however, could have caused muscle shortening and changes in muscle pennation angle, thus affecting the sensitivity of muscle spindles and other mechanoreceptors, altering the H reflex. It has been well established that muscle lengthening and stretching can dramatically influence spinal neural pathways (Budini and Tilp, 2016) because of the activation of the muscle spindle. However, this cannot explain the depression of H reflex registered in the ANKLE condition, where the muscles were not directly compressed. A possible mechanism explaining the drop of H reflex during tissue flossing around the ankle could be the activation of Ib afferents from the Golgi tendon organ (GTO). Even though GTO is usually activated when large pulling forces are exerted to the muscle-tendon complex (i.e., during landing or at large-amplitude stretches) (Guissard and Duchateau, 2006), tissue flossing around the ankle joint could have directly compressed the muscle-tendon junction of the Achilles tendon. Thus, there is possible that some inhibition could arise from the activation of Ib afferents.

In addition, the wrapping pressures in this study were substantially higher compared to previous studies, where an ankle (Driller and Overmayer, 2017) or muscle technique (Kaneda et al., 2020; Konrad et al., 2020) were used. Differences in pressures among studies can arise from several factors such as sensor placement, pressure monitors and flossing technique. As noted by Vogrin et al. (2020a) different wrapping pressures might lead to different physiological responses. They found a statistically significant improvement in knee extensors MVC after a medium wrapping pressure (approximately 140–160 mmHg), but no differences after high wrapping pressure (>200 mmHg). Similar pressure dependency has been recently confirmed using the ankle wrapping technique (Galis and Cooper, 2020). In the present study, there was a negative correlation between wrapping pressure and H reflex change from baseline during the flossing periods (F1–F3), suggesting that the higher the pressure, the greater the inhibition of the H reflex in both ANKLE and CALF conditions. A similar effect was observed in a study, where high-intensity foam-rolling induced a higher H reflex inhibition compared to moderate-intensity and a sham condition (Young et al., 2018). The higher pressure of manual massage led to higher H reflex inhibition, suggesting that higher pressures activate deeper mechanoreceptors (Goldberg et al., 1992).

The results show that the effects of tissue flossing on the H reflex are short-lasting since there were no differences in H reflex 10 min after the intervention. However, the effects may last longer than a few minutes, which was confirmed in the study by Zakutansky et al. (2005) observing that the H reflex was not fully recovered 5 min after acute local ischemia. On the contrary, in a foam rolling study, the soleus H reflex returned to baseline immediately after the pressure was released (Young et al., 2018).

The differences observed at the spinal level did not transfer into functional changes such as higher joint ROM or higher muscle force production. This is surprising since many studies have shown a convincing increase in ROM after joint application (Driller et al., 2017; Driller and Overmayer, 2017) and muscle application (Kaneda et al., 2020); and an increase in MVC after muscle application (Vogrin et al., 2020a; Konrad et al., 2021). In addition, findings from ischemic preconditioning studies suggest that partial or total arterial occlusion leads to improvements in knee extensor voluntary contraction (Paradis-Deschênes et al., 2016b) and higher repeated force capacity in strength-trained athletes (Paradis-Deschênes et al., 2016a). However, the results of this study are well in line with the study conducted by Mills et al. (2020), who found no significant improvements in ROM, jumping and sprint performance in professional rugby union players. As such, the tissue flossing method may be less effective on highly trained individuals.

This study might have several limitations. First, there was a statistically significant difference in the applied pressure of the flossing application between CALF and ANKLE. In this regard, placement of the pressures sensor between both situations needs to be taken into consideration, which in the ANKLE condition was placed directly on a hard structure (cuneiform bones), while in the CALF condition was placed on the muscle aponeurosis between the gastrocnemius bulks (a softer structure). Although the wrapping pressure was monitored in this study, there was no insight into the actual blood-flow occlusion created in the affected leg. To the best of our knowledge, the latter limitation applies to all studies in the field of tissue flossing published up to date. Second, there was a lack of movement while the elastic band was applied, compared to other studies where the participants performed an active ROM movement during the tissue flossing treatment. The participants in the present study were instructed to maintain a steady position in the dynamometer during the flossing intervention so as to measure the H reflex. One can speculate that additional active movement during the flossing intervention, as seen in other studies, play an important role in ROM and MVC improvement. Third, we used a relatively small number of electrical stimuli to evoke the H reflex at each time point. Electrophysiological measurements can be highly variable; thus a large number of stimuli (>20) is usually required to obtain reliable results (Burke, 2016). We were, however, limited by the flossing and rest time (120 s), where we were able to elicit only 10 reflexes at the selected stimulation rate without occurring at the risk of post-activation depression. Finally, to maintain the homogeneity of the group (young elite martial art fighters) we were able to recruit only 11 participants, which represents a limitation to the statistical power of the study.

Even though the majority of the studies investigating the effects of tissue flossing were conducted on young adults, the participants were usually older (>20 years) than the subjects recruited in this study (16 years). It is well known that joint flexibility is age, sex, joint, and movement dependent and decreases with age (Medeiros et al., 2013). However, there is some evidence that junior taekwondo athletes demonstrate lower limb flexibility (sit-and-reach test) scores than most seniors (Bridge et al., 2014). There is a lack of studies comparing the acute effects of stretching methods on adolescents. In a meta-analysis investigating the acute effects of foam rolling on joint flexibility (Skinner et al., 2020), the only study that included adolescent athletes (15 years old) lacked to find significant improvement in ankle ROM after foam rolling (Škarabot et al., 2015). In contrast, other studies, including young adults (>20 years), confirmed that there is a clear beneficial effect of foam rolling on ROM (Skinner et al., 2020). Thus, it is possible that, similarly to other ROM improvement techniques, tissue flossing could affect young athletes differently compared to their older counterparts. As a prospect, it will be interesting to investigate the effects of tissue flossing on different age groups and training statuses.

## Conclusion

Some studies have suggested that tissue flossing could be used as a specific warm-up technique for inducing ROM and explosive strength improvement. However, the findings of this study indicate that high wrapping pressure tissue flossing has a limited influence on joint ROM and plantarflexor MVC in healthy young martial arts athletes. On the other hand, tissue flossing around the muscle has a significant but short-lasting effect on spinal reflex inhibition. The observed changes at the spinal level did not translate into a higher ankle ROM or plantarflexor MVC. Taking into consideration the results of this study, we suggest that tissue flossing is not an advisable technique to be used as a specific warm-up in young elite athletes. The inhibition of the spinal mechanism induced by tissue flossing has to be further investigated to fully take advantage of this method.

## Data Availability Statement

The raw data supporting the conclusions of this article will be made available by the authors, without undue reservation.

## Ethics Statement

The studies involving human participants were reviewed and approved by the Medical Ethics Committee of the University Medical Centre Maribor, Slovenia (UKC-MB-KME-6/21). Written informed consent to participate in this study was provided by the participants’ legal guardian/next of kin.

## Author Contributions

MK, SM, and TS contributed to the conception and design of the study. MK, SM, and FŽ carried out the experiment and data collection and organized the database and data analysis. MK performed the statistical analysis and wrote the first draft of the manuscript. SM, FŽ, MV, and TS wrote sections of the manuscript. All authors contributed to manuscript revision, read, and approved the submitted version.

## Conflict of Interest

The authors declare that the research was conducted in the absence of any commercial or financial relationships that could be construed as a potential conflict of interest.

## Publisher’s Note

All claims expressed in this article are solely those of the authors and do not necessarily represent those of their affiliated organizations, or those of the publisher, the editors and the reviewers. Any product that may be evaluated in this article, or claim that may be made by its manufacturer, is not guaranteed or endorsed by the publisher.
